# Triggered Skin Sensitivity: Understanding Contact Dermatitis

**DOI:** 10.7759/cureus.59486

**Published:** 2024-05-01

**Authors:** Haris M Sheikh, Roshan K Jha

**Affiliations:** 1 College of Medicine, Jawaharlal Nehru Medical College, Datta Meghe Institute of Higher Education and Research, Wardha, IND; 2 Biochemistry, Jawaharlal Nehru Medical College, Datta Meghe Institute of Higher Education and Research, Wardha, IND

**Keywords:** dertits\, dermitis, contact dermitits, irritant contact dermatitis, contact dermatitis

## Abstract

Dermatitis, the incendiary reaction of the skin to various components, can manifest in various types, including atopic dermatitis, contact dermatitis, nummular eczema, seborrhoea, and stasis dermatitis. Atopic dermatitis is the most common skin disease in children and has a growing prevalence in recent years. It is characterized by extreme tingling, eczemous skin injuries, dryness of the skin, and a family history of atopic illnesses. Contact dermatitis (CD) is a common, irritating skin disorder caused by allergens and aggravating elements in the environment. It is the most common cause of work-related dermatitis and plays a substantial role in hand and face dermatitis. A complete restorative history is essential for establishing CD and identifying the allergies that cause it. Fix testing, skin tests for fast contact reactions, serum allergen-specific IgE testing, subjective and quantitative evaluation of allergens inside probable items patients were exposed to, and challenge testing are among the other diagnostic techniques. To avoid a breakdown and the continuation of the skin illness, early and suitable therapy is critical. Allergic dermatitis to contact (ACD) develops during the normal, delayed incendiary reaction and has a perplexing etiology. Accurate identification of the allergen that is causing the reaction allows for adequate individual avoidance. The major treatment alternatives continue to be corticosteroids. Nickel-contact dermatitis is an allergic reaction that affects both children and adults. Adverse contact dermatitis (ACD) is a frequent skin reaction to a common allergen that can affect both children and adults. Less than 10% of all diagnostic procedures in pediatric patients involve checking for ACD symptoms. To answer the clinical question, a thorough history is gathered based on appearance, age group, and dermatitis type. According to pediatricians in the US, metals, perfumes, topical antimicrobials, excessive chemicals, and fabric softeners are the most typical allergens.

## Introduction and background

Dermatitis, the incendiary reaction of the skin to different components, may display an assortment of types. (a) atopic dermatitis, (b) contact dermatitis, (c) nummular eczema, (d) seborrhoea, and (e) stasis dermatitis, conditions with which the persistent may display to the crisis division, either within the intense organization or with the compounding of an unremitting condition, are surveyed in this article [1]. Atopic dermatitis is the foremost common skin malady in children, and its predominance has relentlessly expanded over the last three decades [2]. Atopic dermatitis may be a sort of skin inflammation decided by a polygenic legacy that is frequently related to assisting phenotypes of the atopic disorder, such as outward asthma and unfavorably susceptible rhino conjunctivitis [3]. Atopic dermatitis is a critical appearance of the atopic diathesis with an expanding rate. The normal lifetime prevalence in Switzerland is approximately 13%. The skin infection is characterized by extreme tingling, eczematous skin injuries with regular particular dissemination, dryness of the skin, and an individual's family history of atopic illnesses [4]. One of the most prevalent invasive skin disorders is contact dermatitis (CD), which comprises photoallergic contact dermatitis, relapsing contact dermatitis, light-enhanced contact dermatitis (also called phototoxic contact dermatitis), and proteinaceous contact dermatitis. [5]. Nummular dermatitis (nummular dermatitis) is a kind of dermatitis. Discoid (nummular) skin irritation may be a common and distinct dermatitis variant that has not been thoroughly studied. Even though the administration criteria are equivalent to those of classic atopic dermatitis, adjustments are being made due to its intriguing introduction and meticulous clinical course in youngsters [6]. Unknown in its genesis, seborrheic dermatitis can be a common, regressive, and provocative skin condition. Yeast can help Malassezia grow.

Seborrheic dermatitis frequently affects the scalp, nasolabial folds, glabella, eyebrows, facial hair, ears, retro-auricular skin, sternum, and other skin folds when sebum production is excessive. Seborrheic dermatitis symptoms can be quite severe in those with pigmented skin. In usual contact areas, those with darker skin may have rough, hypopigmented patches and blemishes. Petaloid seborrheic dermatitis, often known as curved or petal-like patches, can develop. Children of color often do not develop the classic "cradle cap" of seborrheic dermatitis, but rather redness, cracking, and hypopigmentation in the afflicted regions and skin folds [7]. The likelihood of developing stasis dermatitis increases with age. It results from reflux-induced venous hypertension brought on by venous valve failure, valve pulsations, or vena cava blockage. Metalloproteases, which are accelerated by iron-containing particles from extravasated red blood cells, induce tissue alterations. Lesions on the lower legs that are itchy and poorly defined, frequently affecting the recognizable ankle, are the initial signs of stasis dermatitis. This is but one of the several skin conditions brought on by chronic venous insufficiency. [8]. Cellulitis, contact dermatitis, and pigmented purpuric dermatoses are among its imitators. Fiery forms are responsible for many of the widespread negative effects of SD [9].

## Review

In this piece, we will look at frequently used terminology connected to these high-risk professions. Surprisingly, there is still a lot of variation in clinical practice, particularly when it comes to topics like bathing, hydration, topical drugs, and hypersensitivity during delivery. Loyal, intimate, and crucial caretakers may become frustrated by the advertisement's anomalies in the assumption and treatment method, as well as its tenacity and persistence [10]. When creating a CD, it is critical to have a comprehensive recovery history, including the history linked to the words. It can supply a CD as well as a list of potential compounds. Aside from the well-known patch test, several additional demonstrative tests may be used to assess CD and identify causative allergens, such as the shrink photo patch test, skin tests to detect quick contact responses, and serum allergens. Specific IgE testing, subjective and quantitative allergy testing of suspicious items to which the patients were exposed, and exposure testing are all available. Recently detected irritants or allergies should be maintained at a safe distance from the therapy. When creating a CD, it is critical to have a detailed restoration history, including the history of the lyrics. It can create a CD with a list of potential chemicals. In addition to the well-known patch test, a variety of evidence tests, such as the fading light test, skin testing for quick contact responses, and serum allergens, could also be used to assess CD and identify causal allergens. Specific IgE testing, both subjective and quantitative, is available for allergies to suspected things to which patients have been exposed as well as exposures. Irritating or allergic substances that have recently been found should be avoided throughout therapy [11].

Allergic contact dermatitis

ACD has an intriguing pathophysiology that takes place during a typical, delayed inflammatory response. Even though a skilled physician may properly evaluate ACD based on its introductory, classic history, and introduction, elective analysis should be considered and avoided. The extreme corroborative test of ACD is fix testing conducted with a significant board of contact allergens. Accurately identifying the triggering allergen allows for appropriate individual avoidance. Corticosteroids remain the primary therapeutic option [12].

Nickel Allergic Contact Dermatitis

Because nickel was used in more consumer goods during the 20th century, nickel sensitivity grew. Ear piercings were fashionable in the 1980s, in contrast to the 1970s craze with buttons, zippers, and studs. In the 1950s and 1960s, shelves were widely used. When nickel-sensitive people are exposed to high amounts of nickel, they often develop nickel-sensitive contact dermatitis and hand dermatitis. In 1990, the Danish government started putting restrictions on consumers' exposure to nickel in response to the growing issue of nickel sensitivity. The EU nickel mandate from 1994 was built on the principles of the Danish and Swedish nickel directives. The research on disease transmission linked to nickel sensitivity was anticipated to change as a result of these significant open health mediators [13].

Children's Clinical Traits and Common Contact Allergies

Both children and adults can develop adverse contact dermatitis (ACD), a severe skin reaction to common allergens. However, only approximately 10 percent of the children's landing tests show signs of ACD. The relative frequency of children's landing attempts may be explained by the drawbacks of testing this group, which include limited space for managing experimental settings and sustaining help during testing, especially with more children. Atopic dermatitis and contact dermatitis flare-ups are two frequent childhood skin diseases that can mimic ACD and cause problems in young adults. After collecting a detailed history and analyzing the frequency, age range, and spectrum of dermatitis, the comparison creates a clinical query. Such a clinical inquiry and persistent dermatitis and a rash with an irregular distribution as additional symptoms are two of the most crucial justifications for doing tests. The US Department of Pediatrics states that the main allergens include metals, perfumes, topical antimicrobials, various compounds, and emollients. Understanding these patterns is crucial for coordinating the staging and precise diagnosis of ACD since it tends to advance in the absence of an allergen and can adversely affect patients' quality of life [14]. The gold standard of care for identifying negative contact dermatitis is testing. Children must undergo landing tests that are tailored to their needs since the allergens that cause their symptoms differ from those that do in adults. Dermatologists might use the Pediatric Design Course of Action, which was created in 2018 as a result of expert consensus and pulls together significant pediatric allergens to assist patients. Deposition testing challenges are particularly challenging to overcome in pediatric patients, such as the requirement for repeated office visits, the length of the deposition application, and the necessity to prevent perspiration and water in the testing location. There are several options presented. Options like open application testing and empiric allergen avoidance, in addition to formal exposure testing, may be advantageous for young people. The key to managing unpleasant contact dermatitis is avoiding allergens, and this emphasizes the necessity to precisely identify the allergens that are responsible for the illness. Continuous data gathering through records is permitted to better comprehend the conclusion and structure of Foot Care Adverse Unaided Contact Dermatitis [15].

Identifying the causes of contact dermatitis

When the skin comes into contact with an outside substance, contact dermatitis develops. Possible causes include touch, airborne particles, odors, and light. Every age group may be impacted. The two most prevalent varieties are intensification contact dermatitis (ICD) and adverse sensitivity contact dermatitis (ACD). ICD is more typical and has more fallacious presumptions. Two more, less typical kinds of contact dermatitis are protein contact dermatitis and food processing photosensitivity. Dermatitis can develop as CD due to coordinated pathway activation without preceding sensitization. In typical ACD, class 4 cell-mediated resistance is not present. Sensitization happens after 5 to 16 days of skin contact with the potential allergen, although there is no acute pain. When you go to exhibits and places with plenty of possible allergens, your chance of sensitization rises. If the dermatitis is persistent, recurring, or affects a person who has never had dermatitis before, contact dermatitis should be taken into consideration. The dorsal corners of the hands are most frequently affected by ICD, most frequently by joining the finger webs. A combination of water, detergents, and cleaning products is the most common cause of ICD, affecting the hands more often than anywhere else. Nickel, fragrances, elastic accelerators, and biocides are the most common sensitizers for ACD. Patients with leg ulcers and stasis dermatitis especially develop hypersensitivity to topical medications, dressings, and gauze. If ACD is suspected, silence should be indicated for corrective testing. Age should not be a barrier to testing fixes. Accurate findings, avoidance of known allergens, and certainty of exacerbation are keys to successful treatment [16].

The Function and Identification of Allergic Contact Dermatitis in Atopic Dermatitis Patients

Patients with atopic dermatitis (AD) may develop atopic contact dermatitis (ACD) as a result of increased allergen exposure, immune system abnormalities (including abnormal cytokine pathways), frequent use of emollients, and topical treatments. Although its validity is still debatable, ACD is a significant clinical issue in both children and adults with Alzheimer's disease [17]. Here, we examine instances in medicine where clinical trials are encouraged. We also go through the restrictions, optimum patch test environment, possible blunders, and difficulties in evaluating the significance of positive results in AD patients [18].

Diagnosis and Management of Contact and Primary Irritant Dermatitis of the Nail Unit

Nail-contact dermatitis is a typical occurrence. The four allergens that are most frequently encountered are toluene, acrylates, and ethyl cyanoacrylate. An adequate clinical history focused on nail grinding is necessary for the evaluation of contact dermatitis, as well as a thorough awareness of common allergens and patch testing. It discusses common allergens and irritants that impact the nail unit. The scalp, palms and soles, and nails are problem areas in standard psoriasis therapy. The pathophysiology, alternative conclusions, and most current therapies are covered in this article [19].

Treating cosmetically-induced nail problems

The $6 billion nail industry is supported by the belief that neat, glossy nails are a sign of success and quality in our culture. Although many women may wear nail polish without experiencing any side effects, it is still crucial to identify the causes and address the issue as soon as it manifests. The key to preventing issues with nail repair is education [20]. Despite these efforts, nail products and practices might result in nail problems that must be recognized and treated to restore nail health [21].

Allergen exposome among different atopic phenotypes

Climate alteration and exposure to natural toxins play a key role in the onset and disturbance of unfavorably susceptible infections. As distinctive climate-dependent designs of atomic immunoglobulin E (IgE) reactivity have been territorially portrayed, we looked to explore the advancing allergen exposome in unmistakable, unfavorably susceptible phenotypes and subtropical climate conditions through an Accuracy Sensitivity Atomic Determination show. Concurrent sensitization to a few houses of tidy vermin (HDM) and capacity vermin molecules was broadly overwhelming within the explored cohort. House tidy bug (HDM) sensitivity has a place among the foremost vital sensitivities and influences around 65-130 million individuals around the world. Furthermore, untreated HDM sensitivity may lead to the improvement of extreme illness appearances such as atopic dermatitis or asthma. Conclusion and immunotherapy for unfavorably susceptible HDM patients are well built up but are regularly hampered by the use of bug extricates that are of awful quality and need critical allergens [22]. Other allergens can cross-react with hemocyanin, tropomyosin, arginine kinase (AK), glutathione S-transferase (GST), and other pan-allergen proteins. In AR patients sensitized to HDM, the use of an atomic or compound solution is advised to identify genuine or cross-reactive sensitization. The effectiveness of HDM immunotherapy in the management of related cross-reactivity in AR patients sensitized to HDM is still debatable and may be influenced by the degree of similarity between the two allergens. In addition, targeting tropomyosin as a potential treatment for HDM patients with allergen cross-reactivity [23]. Understanding the design of IgE sensitization in dermatology-phugoids-allergic patients living in different geological zones is vital to planning an item appropriate for around-the-world allergen immunotherapy (AIT) [24].

Occupational hand dermatitis

Contact dermatitis (CD) is the most prevalent inflammatory skin condition that is made worse by environmental factors and allergens. Atopic dermatitis, the primary reason for rashes on the hands and face, is another typical adverse effect. Other subtypes of contact dermatitis, such as immediate skin reactions, photoinduced contact dermatitis, systemic contact dermatitis, and non-eczema contact dermatitis, have been identified in addition to the two main types of contact dermatitis: toxic sensitized contact dermatitis and aggravating contact dermatitis. A few of the skin disorders that CD is adept at mimicking are atopic dermatitis, lichen planus, and angioedema. In this piece, we'll look at frequently used terminology connected to these high-risk professions. Surprisingly, there is still a lot of variation in clinical practice, particularly when it comes to topics like bathing, hydration, topical drugs, and hypersensitivity during delivery. Loyal, intimate, and crucial caretakers may become frustrated by the advertisement's anomalies in the assumption and treatment method, as well as its tenacity and persistence [25]. ICD and ACD have comparable clinical symptoms, although they have different etiologies and levels of skin occlusion. The fundamental triggers of various forms of metabolism and the activation of innate immunity, which serves as an immune system without a distinct immune system, are irritation of the skin's periphery in ICD [26]. In the sensitization phase, damage to the skin's border can stimulate the innate flagellar pathways, which are required to activate the varied reactivity of T cells in ACD, a contact reaction brought on by type IV contact allergens. Epidermal impedance might thereby raise the risk of ICD or ACD not only by allowing more irritants and allergens to enter the skin but also by delaying the emergence of the intrinsic safe flag required to accept the unfavorable reaction. To prevent CD, it is important to maintain a safe distance from occlusive skin lesions at work. This review focuses on the skin border, how skin injury affects it, and how damage to the border affects ICD and ACD [27].

Identifying and treating contact dermatitis

Inflammatory skin disease, also known as contact dermatitis, is characterized by skin lesions that are red and itchy and are brought on by contact with an external substance. There are two varieties of contact dermatitis: sensitive and aggravated. Contact dermatitis gets worse when a skin-modifying chemical aggravates skin that hasn't been altered [28]. Conversely, sensitized contact dermatitis can be a delayed excessive contact reaction caused by contact with the skin of a foreign substance; exposure to the substance causes skin changes. The most common triggers of contact dermatitis are perfumes, nickel, and poison ivy. The most common symptoms of contact dermatitis are well-defined redness and scaling. In addition, tingling and irritation may occur [29]. In severe situations, emotional energy may appear as redness, blisters, and blisters; lichens may have fissures and cracks. The first stage in verifying a result after determining a plausible cause is determining whether avoiding the drug will fix the issue. A moderate-to-strong topical steroid, such as triamcinolone 0.1% or clobetasol 0.05%, is beneficial in treating localized severe and unfavorable-sensitive contact dermatitis [30]. When negative-sensitivity contact dermatitis affects a significant portion of the skin (more than 20%), systemic steroid treatment is frequently required and offers relief within 12 to 24 hours. Prednisone oral dosage should be progressively reduced over two to three weeks in individuals with a severe rhus rash since abrupt steroid withdrawal can lead to a rebound rash. When a therapy does not work, and the cause or particular allergen is unknown, remedial measures must be taken. More than 6 million chemicals are thought to be present in the environment, of which roughly 3,000 are known to be sensitizers [31]. A cautious operator might be identified by its cautious past. Contact dermatitis may be a minor, self-limiting condition if the causative component is found and eliminated. If the application is not discontinued, skin diseases, including itching, stinging, and other symptoms develop, permanently changing the skin. Identification of an attacker depends on several factors, including background information, word exposure quantities, and the speed with which a rash spreads. In more challenging circumstances, a more thorough investigation, which may involve patch testing, may be required to pinpoint the offender [32]. This article audits treatment, counting antihistamines, topical and verbal steroids, physical measures such as cold-water compresses, and the treatment of auxiliary contamination [33].

## Conclusions

In conclusion, contact dermatitis is a common skin ailment that develops whenever the skin comes into contact with something that makes it itch or react negatively. Swelling, redness, tingling, and, in extreme situations, bleeding might be the symptoms of this. The whole differential diagnosis from the clinical beginning must be known by the doctor treating a patient with an "eczema" rash. There is a great need to scrutinize the identification of a "competitor" who has been hurriedly identified and who does not react to "appropriate" treatment. Skin biopsies are only used to confirm their eczematous (spongiotic) etiology and to rule out other illnesses. Increasing in value, the paradox of fix testing, specifically the tricky effortlessness of application versus the desired skill for elucidation and acknowledgment of clinical importance, is the key to the correct administration of the understanding of contact dermatitis. The most efficient method of treating contact dermatitis is to identify and stay away from the allergen or triggering substance. This could entail adapting one's lifestyle to avoid specific pollutants, using protective gear, and using sensitive skin care products. In a few cases, restorative treatment may be fundamental to reducing indications and advancing healing. It’s critical to note that contact dermatitis can shift in severity and duration, with a few cases settling rapidly, whereas others may require progressive administration. Furthermore, a few people may be more inclined to develop contact dermatitis due to their hereditary inclination or occupation. Anticipation plays a pivotal role in overseeing contact dermatitis, and people ought to be proactive in distinguishing potential triggers and taking essential safety measures. Counseling with a dermatologist or healthcare professional is suggested for a precise determination, personalized treatment plan, and direction on preventive measures. By understanding the causes, symptoms, and administration methodologies of contact dermatitis, people can minimize the effects of this condition on their everyday lives and maintain solid, comfortable skin.
